# Tunable Superconductivity in BSCCO via GaP Quantum Dots

**DOI:** 10.3390/ma18235458

**Published:** 2025-12-03

**Authors:** Qingyu Hai, Duo Chen, Ruiyuan Bi, Yao Qi, Lifeng Xun, Xiaoyan Li, Xiaopeng Zhao

**Affiliations:** Smart Materials Laboratory, Department of Applied Physics, Northwestern Polytechnical University, Xi’an 710129, China; haiqingyu@mail.nwpu.edu.cn (Q.H.); chenduo@mail.nwpu.edu.cn (D.C.); biruiyuan@mail.nwpu.edu.cn (R.B.); qiyao@mail.nwpu.edu.cn (Y.Q.); xunlifeng@mail.nwpu.edu.cn (L.X.); lixiaoyan0521@mail.nwpu.edu.cn (X.L.)

**Keywords:** B(P)SCCO, GaP quantum dots, electroluminescent, heterophase, injecting energy, smart superconductivity

## Abstract

The enhancement of superconducting properties of high-temperature copper-oxide superconductors like B(P)SCCO remains a hot research topic in the field of superconducting materials. This study introduces GaP quantum dots (QDs) as a heterophase, leveraging their electroluminescent properties to enhance the superconductivity of B(P)SCCO. Experimental results demonstrate that the electroluminescence generated by GaP quantum dots (QDs) under an applied electric field induces tunable superconducting enhancement of B(P)SCCO. A reproducible trend of enhancement in the critical transition temperature (*T_c_*) and depairing current density (*J_d_*) is observed with increasing QD electroluminescent intensity, suggesting a positive correlation. This electroluminescence-induced enhancement dominates over the inherent impurity effects at optimal QD content.

## 1. Introduction

Bismuth-based superconducting materials (general formula: Bi_2_Sr_2_Ca_n−1_Cu_n_O_2n+4+δ_) [1,2,3,4,5], as a representative copper-oxide superconductor, exhibit significant application value in power transmission [6], high-field magnets [7], and energy storage due to their high critical temperature (*T_c_* = 110K for Bi-2223 phase [5]), absence of rare-earth elements, and superior processability. However, the intrinsic limitations of BSCCO such as strong anisotropy caused by their layered structure [1,8], weak interlayer coupling, and insufficient intrinsic flux pinning severely constrain performance in high-current and high-field environments [9,10,11]. Traditional elemental doping (e.g., Pb [12,13], Sb [14,15]) can improve phase purity yet offers limited enhancement in critical transition temperature (*T_c_*) and critical current density (*J_c_*). In contrast, the nanocomposite strategy—introducing heterophase nanoparticles as artificial pinning centers (e.g., insulator Al_2_O_3_ [16], semiconductor SiC [17], ferromagnet FePb [18])—has emerged as an effective modification approach for (Bi,Pb)-Sr-Ca-Cu-O (B(P)SCCO) superconductors [19,20,21,22,23,24,25,26,27,28]. This strategy leverages interface coupling effects between nanoscale heterophases and the superconducting matrix to create strong pinning sites, significantly enhancing flux pinning force density (*F_p_*) and boosting *J_c_* by 1~2 orders of magnitude [29,30,31,32,33]. However, it typically comes at the cost of a suppressed *T_c_* due to impurity effects and interface scattering. Clearly, although this method has been experimentally confirmed as an effective approach to improve the current carrying capacity of superconductors, it still does not represent the optimal strategy for enhancing the superconductivity of B(P)SCCO materials.

Metamaterials [34,35] are artificially engineered microstructures or composite materials that exhibit extraordinary physical properties unattainable in natural materials, characterized by their structure-determined functionalities. Researchers proposed the concept of metamaterial superconductors [36,37,38], aiming to develop superconducting materials with elevated critical temperatures *T_c_* through precisely engineered superconducting architectures. In recent years, the Max Planck Institute proposed light-induced superconductivity [39,40,41,42,43]. Their research team discovered that under mid-infrared laser irradiation, both YBCO, and K_3_C_60_ thin films exhibit transient superconducting behavior. Inspired by the concept of metamaterials, our team proposes a novel modification strategy that incorporates electroluminescent heterophases into superconducting materials. By introducing the electroluminescent heterophase as “meta-atoms” into the superconducting particle “matrix”, we construct a composite structure. The core hypothesis is that photons generated internally via electroluminescence could couple with Cooper pairs, potentially reinforcing the pairing strength and enhancing superconductivity, a system we term a Smart Metamaterial Superconductor (SMSC).

Previous studies demonstrated that although introducing Y_2_O_3_:Eu^3+^/Ag luminescent heterophase enhances superconductivity in MgB_2_ [44,45,46,47,48,49] (Δ*T_c_* = +0.4 K, Δ*J_c_* = +20%) and B(P)SCCO [50,51,52] (Δ*T_c_* = +1 K, Δ*J_c_* = +24%), its performance is severely limited by high electric field dependence, low luminescent intensity, and rapid decay characteristics, compromising the enhancement efficiency of superconductor. In contrast, GaN p-n junction particles exhibit superior advantages including high luminescent quantum efficiency and low turn-on voltage, achieving stronger superconducting enhancement in MgB_2_ [53,54] (Δ*T_c_* = +0.8 K, Δ*J_c_* = +35%) and B(P)SCCO [55] (Δ*T_c_* = +2 K, Δ*J_c_* = +35%). However, their micron-scale dimensions (average ~2 μm) introduce significant impurity effects, which substantially offset the potential superconducting gain from electroluminescence. It is noteworthy that prior research has primarily focused on the influence of heterophase content, leaving the systematic role of the heterophase’s electroluminescence intensity—a key variable in the proposed mechanism—largely unexplored. To address this critical gap and to simultaneously mitigate the limitations of previous electroluminescence phases. we select GaP core–shell quantum dots (QDs) [56] as the electroluminescent heterophase. GaP QDs offer superior stability compared to Y_2_O_3_:Eu^3+^/Ag and a much smaller size (~3.5 nm) than GaN particles. In our preliminary work, incorporating these into MgB_2_ superconductors [57], we observed an increasing trend in both *T_c_* and *J_c_* with enhanced luminescence intensity, providing the initial validation of light intensity as an independent parameter. Specifically, it was found that both critical temperature *T_c_* and critical current *J_c_* density improved as the luminescence intensity of GaP quantum dots increased.

In this study, we synthesized GaP:Zn^2+^/GaP–GaInP–GaP:Te^2−^/GaP core–shell quantum dots [56] with tunable electroluminescent intensities via the thermal injection method. These were incorporated into a B(P)SCCO superconducting matrix to systematically investigate the independent and coupled effects of two variables: the “electroluminescence intensity” and the “addition content” of the luminescent heterophase. This work aims to investigate the potential influence of the heterophase’s electroluminescence intensity on the superconducting properties and to explore a novel modification strategy for B(P)SCCO superconductors.

## 2. Materials and Methods

### 2.1. Synthesis of GaP:Zn^2+^/GaP–GaInP–GaP:Te^2−^/GaP Core–Shell Quantum Dots

**Figure 1:**
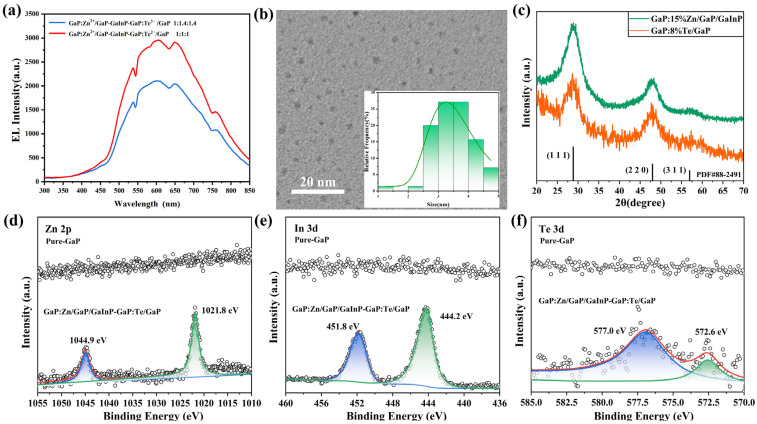
(**a**) EL spectra of GaP quantum dot electroluminescent particles, (**b**) TEM images of GaP quantum dot electroluminescent particles, (**c**) XRD patterns of GaP quantum dot electroluminescent particles, and (**d**–**f**) XPS analysis of GaP quantum dot electroluminescent particles.

In this study, GaP:Zn^2+^/GaP–GaInP–GaP:Te^2−^/GaP core–shell quantum dots (hereinafter referred to as GaP QDs) with tunable electroluminescence intensities were synthesized following the thermal injection strategy reported in Ref. [56]. The synthesis was achieved via a thermal injection approach by modulating the reaction durations of individual layers (GaP:Zn^2+^/GaP, GaInP, and GaP:Te^2−^/GaP). GaP:Zn^2+^ cores were synthesized by rapidly injecting a (TMS)_3_P–TOP solution into a mixture of Ga(acac)_3_, Zn(acac)_3_, oleic acid (OA), and 1-octadecene (ODE) at 300 °C under Ar protection. The GaP active layer was subsequently grown by repeating the hot-injection of (TMS)_3_P, and a GaInP quantum-well shell was deposited by co-injecting Ga(acac)_3_ and InCl_3_ precursors at 300 °C, forming GaP:Zn/GaP/GaInP multilayer QDs. The GaP:Te^2−^/GaP shell layer was synthesized using an analogous procedure, in which Te powder was used in place of Zn(acac)_3_. Figure 1b displays the Transmission Electron Microscopy (TEM) image of GaP quantum dots. The inset shows the corresponding size distribution histogram. The nanoparticles demonstrate uniform size distribution, centered at ~3.5 nm (range: 2.5–4.5 nm) and following a normal distribution. The blurring observed in the imaging primarily originates from the oleylamine ligand layer coating the quantum dot surfaces. The samples underwent X-ray diffraction (XRD) characterization, as shown in Figure 1c. The vertical black lines denote the standard XRD pattern of zinc-blende structured bulk GaP. As evidenced in the figure, the diffraction peaks of our samples exhibit precise alignment with the reference pattern, demonstrating three characteristic peaks corresponding to the (111), (220), and (311) crystallographic planes of the zinc-blende GaP structure. Notably, no discernible impurity peaks were observed. This demonstrates that the synthesized samples consist of zinc-blende-structured GaP quantum dots, with Zn^2+^ and Te^2−^ ions successfully doped into the GaP crystal lattice without detectable elemental segregation or secondary phase formation. Moreover, the absence of XRD peak shifts demonstrates that low-concentration Zn^2+^/Te^2−^ doping induces no detectable lattice strain. Figure 1a displays the electroluminescence (EL) spectrum of the sample under a 7 V bias voltage. The spectrum reveals red emission centered at ~600 nm with a full width at half maximum (FWHM) of ~200 nm under electric field. The 1:1:1 GaP quantum dots (layer-wise reaction time ratio) exhibit a higher emission intensity of ~2950 a.u., while the 1:1.4:1.4 GaP quantum dots show a reduced intensity of ~2100 a.u. Furthermore, we conducted X-ray photoelectron spectroscopy (XPS) analysis to investigate the elemental composition and valence states of the multilayer core–shell GaP nanoparticles. As shown in Figure 1d–f, distinct emission peaks in the Zn 2p, Te 3d, and In 3d characteristic regions confirm the successful doping of Zn^2+^, Te^2−^, and In^3+^ ions into the GaP lattice via the thermal injection method [56].

### 2.2. Preparation of B(P)SCCO Superconducting Samples

In this study, B(P)SCCO superconducting samples were synthesized via solid-state sintering. Stoichiometric quantities of high-purity precursors (Bi_2_O_3_, PbO, SrCO_3_, CaCO_3_, CuO; all Alfa Aesar, 99.99%) were weighed according to the molar ratio of Bi:Pb:Sr:Ca:Cu = 0.9:0.4:2:2:3. To compensate for the potential evaporation of Bi and Pb during the prolonged high-temperature sintering process, a slight excess of these elements was accounted for in the initial weighing. The mixed powder and Φ 5 mm agate balls were loaded into a 50 mL milling jar with anhydrous ethanol as a dispersant. Milling was performed in a planetary ball mill (500 rpm, alternating rotation) for 20 h. The as-milled powder was vacuum-dried at 70 °C for 12 h, then sieved through a 500-mesh sieve to obtain a uniform particle size of 25 ± 3 μm. The raw material powder was calcined in a tubular furnace in air at 840 °C for 50 h (heating rate: 4 °C/min; cooling rate: 2 °C/min, with controlled cooling to 800 °C, followed by furnace cooling). After cooling, the powder was collected and subjected to grinding for one hour using an agate mortar. This thermal-mechanical treatment cycle was repeated twice, ultimately yielding black pre-calcined B(P)SCCO powder.

A measured amount of B(P)SCCO pre-calcined powder was loaded into a mold and compacted under 12 MPa pressure for 10 min to form pellets (Φ 12 × 1 mm). These pellets were then transferred to an alumina boat and sintered in a tubular furnace in air at 840 °C for 30 h (heating rate: 4 °C/min; cooling rate: 2 °C/min with controlled cooling to 800 °C, followed by furnace cooling). After cooling to room temperature, the pellets were removed and ground into B(P)SCCO powder.

To prepare the composite samples, GaP quantum dots with varying electroluminescent intensities were mixed with the B(P)SCCO powder at designated addition ratios (0, 0.15, 0.2 wt.%) by grinding in an agate mortar for 15 min. The mixed powders were then compacted under 12 MPa pressure for 10 min to form pellets (Φ 12 × 1 mm). All composite pellets (S0–S4) were sintered at 840 °C for 120 h in air in a tubular furnace, with the sintering process carefully controlled to ensure uniformity. In this study, the pure B(P)SCCO sample was designated as S0. The sample containing GaP quantum dots without electroluminescence (content: 0.2 wt.%) was designated as S1. The sample with GaP quantum dots added (content: 0.2 wt.%) with an electroluminescence intensity of 2100 a.u. was designated as S2. Samples with GaP quantum dots added (content: 0.15 wt.%, 0.2 wt.%) with an electroluminescence intensity of 2950 a.u. were designated as S3 and S4, respectively, as detailed in Table 1.

### 2.3. Property Characterization of B(P)SCCO Superconducting Samples

The surface morphology of the samples was examined using an FEI Verios G4 ultra-high-resolution field emission scanning electron microscope (SEM) (Thermo Fisher Scientific, Hillsboro, OR, USA). Energy-dispersive X-ray spectroscopy (EDS) was employed to confirm the elemental composition. To investigate phase formation, X-ray diffraction (XRD) analysis was performed on a Bruker D8 Advance diffractometer (Bruker AXS, Karlsruhe, Germany) with Cu-Kα radiation (λ = 1.5406 Å) over a 2θ range of 10–70°. The electrical transport properties of the samples, including the resistance-temperature (*R-T*) relationship for determining the critical temperature (*T_c_*) and the current-voltage (*I*-*V*) characteristics for determining the depairing current (*I_d_*), were measured using the standard four-probe method. First, four indium electrodes, each with dimensions of 1 mm × 1 mm and spaced 1 mm apart, were pressed onto the surface of the sintered samples using the press-contact method. During measurements, the electrodes on the two sides were connected to a Keithley 6221 current source (Keithley Instruments Inc., Cleveland, OH, USA)via enameled copper wires to provide a stable test current, while the two central voltage electrodes were connected to a Keithley 2182A nanovoltmeter (Keithley Instruments Inc., Cleveland, OH, USA) to collect voltage signals. The entire measurement process was conducted in a vacuum low-temperature environment provided by a closed-cycle cryostat manufactured by Advanced Research Systems (Macungie, PA, USA), with a minimum temperature of 10 K. Temperature measurements and control during the experiment were managed by a Lake Shore 335 temperature controller (Lake Shore Cryotronics, Inc., Westerville, OH, USA). The depairing current density (*J_d_*) was determined from current-voltage (*I*-*V*) characteristics measured in zero applied magnetic field at various temperatures, using a 1 μV/cm electric field criterion. The DC magnetization of the sample was measured under a magnetic field of 100 Oe in zero-field cooling mode.

## 3. Results

### 3.1. Phase Analysis

**Figure 2:**
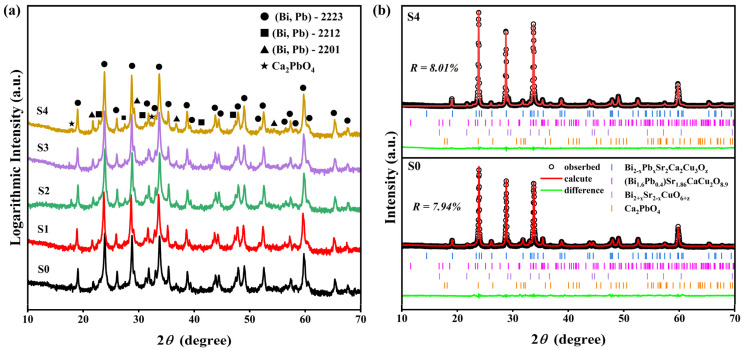
(**a**) XRD patterns of the samples S0 to S4, (**b**) Refined fitting plots for samples S0 and S4.

The phase composition of the samples was characterized by X-ray diffraction (XRD). Figure 2a displays the XRD patterns of samples S0 (pure sample) to S4. To enhance the visibility of minor peaks, the diffraction intensity is plotted on a logarithmic scale on the y-axis. It can be observed that all major diffraction peaks correspond to the (Bi,Pb)-2223 high-temperature superconducting phase, while minor peaks originate from the low-temperature phases of (Bi,Pb)-2212 and (Bi,Pb)-2201. In the patterns of samples S1–S4 with added GaP quantum dots, no diffraction peaks corresponding to GaP or its related oxides (such as Ga_2_O_3_, P_2_O_5_, etc.) were detected, which may be attributed to the low addition content of GaP quantum dots.

Rietveld refinement of the XRD data was performed to analyze the crystallographic parameters of (Bi,Pb)-2223 phase. Figure 2b presents the refinement fitting patterns for the samples S0 and S4. The phase fractions and corresponding lattice parameters, derived from the refinement results, are summarized in Table 2. The data reveal that the content of the dominant (Bi,Pb)-2223 phase remains consistently around 93% across all samples, with no significant variation in lattice parameters. These results indicate that the incorporation of GaP quantum dots does not markedly alter the primary phase composition or crystal structure of the B(P)SCCO matrix.

### 3.2. Microstructural Analysis

**Figure 3:**
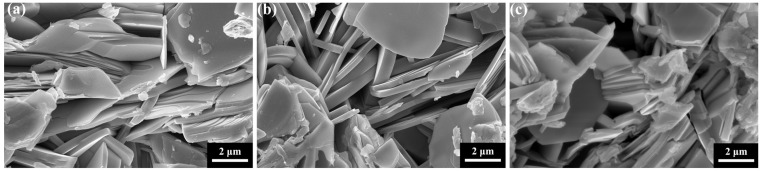
SEM images of samples (**a**) S0, (**b**) S1, (**c**) S4.

Figure 3 presents representative scanning electron microscopy (SEM) images of the pure B(P)SCCO sample (S0) and the GaP quantum dot-doped samples S1 and S4. All samples exhibit the characteristic defined layered morphology typical of B(P)SCCO superconductors, with a uniform distribution of grain sizes. Comparative analysis demonstrates that the incorporation of GaP quantum dots does not disrupt the intrinsic layered grain structure, nor does it introduce observable microstructural defects or abnormal grain growth. This confirms that the heterophase addition preserves the microstructural integrity of the B(P)SCCO matrix.

**Figure 4:**
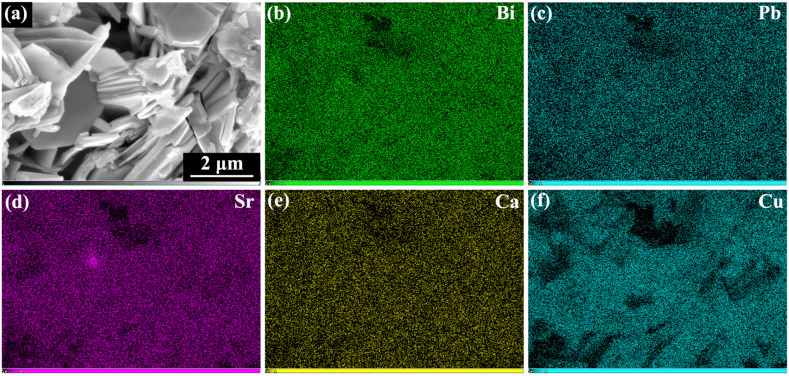
(**a**) BSE image of sample S4 and the corresponding EDS elemental maps for (**b**) Bi, (**c**) Pb, (**d**) Sr, (**e**) Ca, and (**f**) Cu.

To further assess the compositional homogeneity and distribution of constituent elements, backscattered electron (BSE) imaging and energy-dispersive X-ray spectroscopy (EDS) mapping were performed on sample S4. As shown in Figure 4a, the BSE image displays a homogeneous contrast without pronounced brightness variations, suggesting the absence of large-scale elemental segregation or secondary phase agglomeration. The corresponding EDS elemental maps (Figure 4b–f) show continuous and uniform distributions of the principal matrix elements—Bi, Pb, Sr, Ca, and Cu—throughout the scanned region. No localized enrichment or depletion of these elements is detected, confirming the compositional homogeneity of the composite sample at the microscale.

These results collectively confirm that the composite sample maintains compositional homogeneity at the microscale and that the introduced GaP quantum dots do not induce detectable segregation or structural perturbation within the resolution limit of the microscopy techniques employed.

### 3.3. R–T Curve Measurement

**Figure 5:**
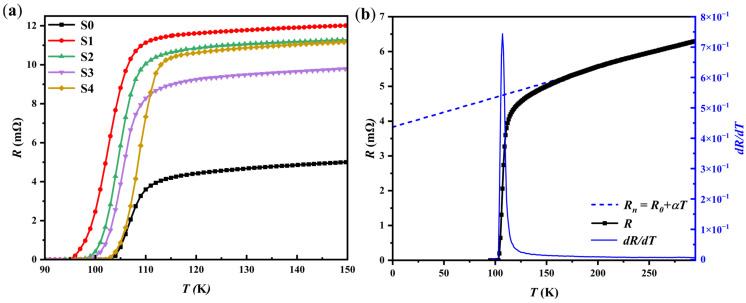
(**a**) The curve of samples’ resistance–temperature variation within the temperature range of 90–150 K, (**b**) the derivatives (*dR*/*dT*) of resistance *R* with respect to temperature *T* at various temperatures (blue solid line), along with the fitted normal-state resistance *R_n_* (blue dashed line) for the samples S0.

The electrical resistance–temperature relationship of the samples was measured over the temperature range of 90–295 K using the standard four-probe method with a current of 1 mA to prevent Joule heating effects. Figure 5a shows the R–T curves of all the samples within the temperature range of 90–150 K. All samples exhibit characteristic high-temperature superconducting transition behavior of copper oxides. Figure 5b presents the resistance-temperature curve for sample S0 and its derivative, *dR*/*dT*. As the temperature decreases, the sample shows strange metal behavior characterized by a linear decrease in resistance, which follows the relation *R*(*T*) *= αT* + *R*_0_. Below the characteristic temperature T*—defined as the temperature at which (*R*(*T*) − *R*_0_)/*αT* begins to deviate from 1—the resistance gradually departs from the linear temperature dependence, showing an upward curvature, while *dR*/*dT* gradually increases. This is generally interpreted as the sample entering the pseudogap state. In this regime, the opening of the pseudogap is accompanied by enhanced electron correlations [58,59], and preformed Cooper pairs begin to emerge, although long-range phase coherence has not yet been established. This phenomenon is commonly observed in underdoped copper-oxide high-temperature superconductors. As the temperature further decreases below the onset transition temperature *T_c,on_*, the resistance drops sharply, accompanied by a steep rise in *dR/dT*, indicating the establishment of global superconducting coherence. When the temperature falls below the zero-resistance transition temperature *T_c,_*_0_, the resistance vanishes completely, and the sample enters a fully superconducting state, in which the Cooper pairs achieve long-range phase coherence. It is noteworthy that a two-step transition is universally observed in polycrystalline high-temperature superconductors [60,61,62,63]. This phenomenon, typically characterized by two distinct peaks in *dR/dT* between the onset transition temperature (*T_c,on_*) and the zero-resistance temperature (*T_c,_*_0_), is commonly attributed to factors such as weak intergranular links and porosity, the presence of excessive amounts of low-*T_c_* superconducting phases, or Joule heating induced by high excitation currents. In contrast, such a two-step transition is absent in the samples of the present work, where only a single, sharp superconducting transition is observed. Combined with XRD and SEM results, the absence of the two-step transition in our samples can be ascribed to the high phase purity of the (Bi,Pb)-2223 superconductor, excellent intergranular connectivity, and the effective suppression of weak links at grain boundaries. Furthermore, the use of a low measuring current effectively avoided any thermal effects that could arise from current flow.

The superconducting transition intervals for samples S0 to S4 are 103–114, 95–112, 97–113, 98–114, and 102–114, respectively. In this study, the critical transition temperature *T_c_*, is defined as the temperature at which the value of *dR/dT* reaches its maximum. The parameters extracted from the sample *R–T* curves, including *T_c,_*_0_, *T_c,on_*, *T_c_*, and *R*_295*K*_ are summarized in Table 3. As shown in Table 3, the introduction of GaP quantum dots (S1–S4) leads to increased room temperature resistance (*R*_295*K*_) and reduced *T_c,_*_0_ compared with the pure sample S0. This trend reflects the impurity scattering associated with the heterogeneous GaP phases, which affects the normal-state charge transport and weakens superconducting coherence at low temperatures. Overall, the impurity effect induced by the introduction of GaP quantum dots leads to a reduction in the superconducting transition temperature. However, among samples S1, S2, and S4 with the same nominal heterophase addition level (0.2 wt.%), the critical temperature *T_c_* shows a gradual recovery as the electroluminescence intensity of the GaP quantum dots increases. Furthermore, for samples with similar electroluminescence intensity but different addition amounts, S3 (0.15 wt.%) and S4 (0.2 wt.%), the sample with the higher addition content (S4) exhibits a higher *T_c_*. These results collectively demonstrate that the electroluminescence from the GaP quantum dots enhances the superconducting properties of the samples, and this effect exhibits a positive correlation with both the luminescence intensity and the addition content.

### 3.4. J_d_–T Curve Measurement

The current-voltage (*I-V*) characteristics of the samples were measured using the standard four-probe method at various temperatures. To eliminate thermal effects and thermoelectric voltages induced by the measuring current, bipolar pulsed currents were employed. Under zero-field conditions, when the current passing through the sample exceeds a certain critical value, the superconducting state is disrupted, and the sample exhibits normal-state behavior; this current is defined as the depairing current *J_d_* [64,65]. The depairing current *I_d_* for each sample was determined based on a quench criterion of 1 μV/cm, from which the depairing current density *J_d_* was calculated.

**Figure 6:**
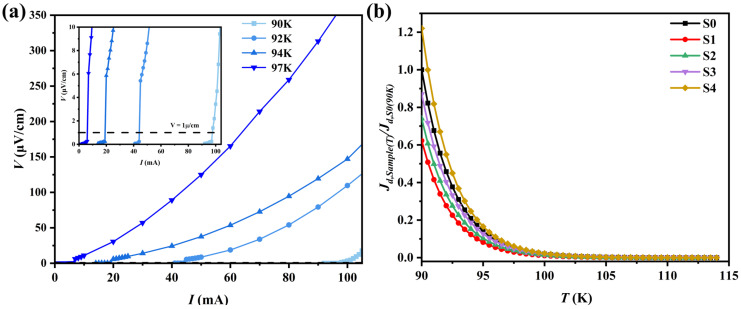
(**a**) *I-V* curves of sample S4 at different temperatures, (**b**) depairing current *J_d_* of the samples S0 to S4 within the temperature range of 90–115 K.

Figure 6a displays the *I-V* characteristics of sample S4 at different temperatures, As shown in Figure 6b, all samples exhibit typical characteristics of B(P)SCCO superconductors, namely a gradual decrease in *J_d_* with increasing temperature until the superconducting state completely vanishes. Compared to the pristine sample S0, samples S1–S3 with introduced GaP quantum dots show a more pronounced suppression of *J_d_*, which is primarily attributed to the impurity effect resulting from the incorporated heterogeneous phases. This effect reduces the superconducting transition temperature *T_c_*, thereby causing the samples to enter the superconducting-to-normal transition region at an earlier stage.

It can be observed that among samples with the same heterophase addition content (0.2 wt.%), *J_d_* exhibits a systematic increase with enhanced electroluminescence intensity of the GaP quantum dots (from S1, S2 to S4). Furthermore, comparing samples S3 (0.15 wt.%) and S4 (0.2 wt.%) with similar luminescence intensity but different addition amounts reveals that S4, with the higher addition content, demonstrates superior *J_d_* performance, achieving an improvement of approximately 20% compared to S0.

These results indicate that the electroluminescent properties of GaP quantum dots exert an enhancing effect on the current-carrying capacity of B(P)SCCO superconductors. This enhancement not only correlates positively with the electroluminescence intensity but is also influenced by the addition content.

### 3.5. DC Magnetic Magnetization Measurement

**Figure 7:**
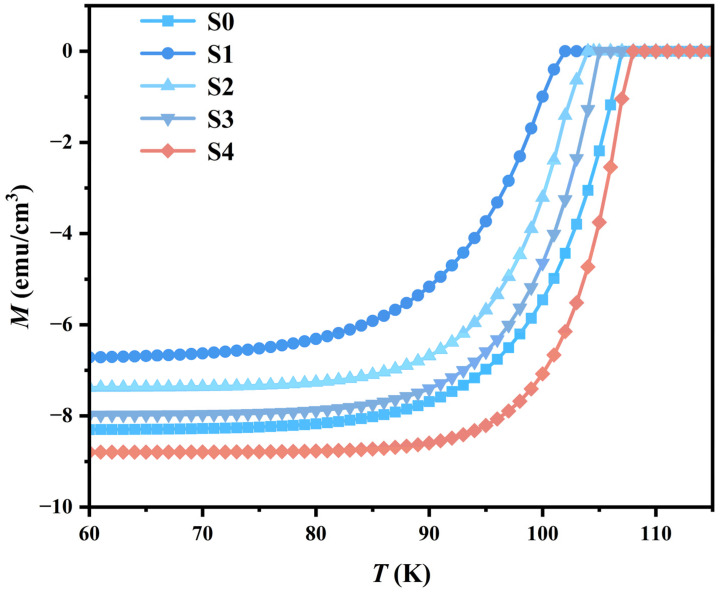
The DC magnetic magnetization of the samples S0 to S4.

Figure 7 presents the temperature dependence of DC magnetization (*M*-*T*) for all samples, measured in zero-field-cooled (ZFC) mode under an applied magnetic field of 100 Oe. The mass magnetization data (in emu/g) were converted to volume magnetization (in emu/cm^3^) using the geometrically measured density of the pelleted samples. All samples exhibit a clear diamagnetic response upon cooling, signifying the onset of superconductivity. The magnetization gradually weakens with increasing temperature until it approaches the normal-state value, which is consistent with the behavior of B(P)SCCO superconductors. The temperature at which the diamagnetic signal vanishes—defined as the point where the *M-T* curve merges with the normal-state baseline—is identified as 107 K, 102 K, 104.5 K, 105 K, and 108 K for samples S0, S1, S2, S3, and S4, respectively. Compared to the superconducting transition region revealed by the electrical transport measurements in Figure 5, the transition region displayed in the magnetization measurements is notably broader. This discrepancy is primarily attributed to their distinct underlying physical processes: the resistive transition marks the establishment of a zero-resistance pathway, which emerges as soon as a percolating superconducting network forms. In contrast, the magnetic transition reflects the gradual development of the bulk superconducting state (the Meissner effect), a process that can be hindered by factors such as grain boundaries, weak links, and defects.

Notably, among the samples with an identical heterophase addition content of 0.2 wt.%, the diamagnetic vanishing temperature shows a systematic increase with the enhanced electroluminescence intensity of the GaP quantum dots (progressing from S1 to S2 to S4). Furthermore, a comparison between samples S3 (0.15 wt.%) and S4 (0.2 wt.%), which possess similar luminescence intensity, reveals that S4 with the higher addition content exhibits a superior performance in the characteristic magnetic onset transition temperature (*T_m,onse_*_t_). These magnetic measurements are in good agreement with the electrical transport results. Together, they suggest a systematic tunability of the superconducting properties in B(P)SCCO upon the introduction of GaP quantum dots and indicate a measurable correlation between the electroluminescent characteristics of the dopants and the resultant superconducting parameters.

## 4. Discussion

This study systematically investigates the influence of incorporating gallium phosphide (GaP) quantum dots with varying electroluminescent characteristics on the superconducting properties of B(P)SCCO. The results indicate that the introduction of GaP quantum dots exerts a dual, competing influence. On one hand, the quantum dots themselves, as a heterogeneous phase, inevitably cause an impurity effect that suppresses superconductivity; on the other hand, their electroluminescence may induce a certain enhancement.

Specifically, sample S1, with the introduction of non-electroluminescent GaP quantum dots, exhibits a reduction in both the critical temperature (*T_c_*) and the depairing current density (*J_d_*) compared to the pure sample S0. This observation directly manifests the impurity effect arising from the incorporated heterogeneous phase. However, as the electroluminescence intensity of the added quantum dots increases (samples S2, S3), a gradual recovery in both *T_c_* and *J_d_* values is observed. Notably, for sample S4, which combines a higher luminescence intensity with a greater addition content, the zero-resistance transition temperature (*T_c,_*_0_) approaches that of S0, the onset transition temperature (*T_c,on_*) matches that of S0, and both the critical temperature (*T_c_*) and depairing current density (*J_d_*) surpass the values of the pure sample. Although, constrained by the electroluminescence intensity of the GaP quantum dots, the *T_c,_*_0_ and *T_c,on_* of the samples with added GaP QDs do not exceed those of the pure sample and the enhancement in *J_d_* remains limited, this systematic variation unequivocally demonstrates that the electroluminescent property of the GaP quantum dots exerts a measurable positive influence on the superconducting performance. Furthermore, this positive effect exhibits a clear positive correlation with both the electroluminescence intensity and the addition content.

Integrated characterization results from XRD and SEM confirm that the introduction of GaP quantum dots does not significantly alter the primary phase structure, microstructure, or elemental distribution of the B(P)SCCO matrix. Based on this finding, we compare its effect with that resulting from the addition of conventional non-electroluminescent nanoparticles. The latter typically enhance the depairing current density (*J_d_*) by introducing defects for flux pinning, but often suppress the critical temperature (*T_c_*) due to concomitant impurity scattering, representing a case of “single-effect modulation.” In contrast, the luminescent GaP quantum dots introduced in this work demonstrate the potential for “dual-effect enhancement,” characterized by a concurrent improvement in both *T_c_* and *J_d_* with increasing electroluminescence intensity. This distinction suggests that the electroluminescence property likely governs a physical process distinct from conventional defect-mediated flux pinning.

Building on our previous investigations into light-emitting heterophases as a means to modulate superconducting properties, we further explore the underlying physical mechanisms and propose a preliminary theoretical model. We posit that, under an external electric bias, photons generated by electroluminescence within the heterophase may weakly couple to Cooper pairs in the superconductor. Specifically, such localized optical fields can excite surface plasmons and generate evanescent electromagnetic waves, which in turn modulate the energy matching and transport pathways of carriers at the superconductor’s surface or interface, thereby moderately enhancing the Cooper-pairing interaction [54,66]. This evanescent-field-mediated coupling is expected to exhibit an intensity-dependent resonant enhancement, consistent with our experimental observation that both *T_c_* and *J_d_* increase concurrently with the electroluminescence intensity.

Beyond this “evanescent-field–Cooper-pair coupling” mechanism, numerous studies have shown that optical or high-frequency electromagnetic fields can enhance superconductivity through several distinct pathways. First, non-equilibrium quasiparticle redistribution (Eliashberg mechanism) [67,68], in which an alternating field drives the quasiparticle distribution *f*(E) away from equilibrium, transiently increasing the superconducting gap Δ and *T_c_*. Second, coherent excitation of the order-parameter amplitude mode (Higgs mode) [69,70], whereby strong THz fields resonantly drive the amplitude mode at ℏω ≈ *2*Δ and momentarily reinforce the superconducting order. Third, optically induced nonlinear lattice dynamics and suppression of competing phases. For example, in the stripe-ordered cuprate La_1.675_Eu_0.25_Sr_0.125_CuO_4_, resonant excitation of the Cu–O stretching mode by the optical field [39] suppresses the stripe phase that competes with superconductivity and restores interlayer Josephson coupling, thereby inducing a transient superconducting state. A similar mechanism has been reported in K_3_C_60_, where optical-field-driven excitation of infrared-active phonons triggers nonlinear phonon coupling and consequently enhances the electron–phonon interaction [41]. In addition, interface-related enhancement effects [71,72], widely observed in nanoscale heterostructures, may also contribute in our system: variations in the local dielectric environment, strain state, or interfacial phonon spectrum can subtly modify the pairing interactions and thus elevate the superconducting performance.

Although the local optical fields generated by GaP quantum dots are far weaker than those produced by intense THz or mid-infrared pulses, the associated perturbations—localized electromagnetic fluctuations, slight interfacial charge rearrangements, and modest modifications of the vibrational and phononic environment—may still trigger weak non-equilibrium responses or subtle adjustments to the local pairing landscape. The cooperative action of these microscopic effects may account for the moderate enhancements in *T_c_* and *J_d_* observed in our experiments. The positive correlation between *T_c_* and electroluminescence intensity further supports this integrated theoretical picture. In conclusion, the findings of this study demonstrate that the introduction of electroluminescent GaP quantum dots can induce an enhancement effect in B(P)SCCO superconductors, likely related to photon–Cooper pair coupling. This beneficial effect competes with the inherent impurity effect introduced by the quantum dots, and their interplay collectively governs the final superconducting performance. Although the precise microscopic mechanism requires further elucidation through additional experiments and refined theoretical modeling, the phenomena revealed in this work provide a valuable experimental foundation for exploring novel interactions between photons and the superconducting state.

## 5. Conclusions

This study investigates the effect of the electroluminescence intensity and addition content of GaP quantum dots on the superconducting properties of B(P)SCCO, including critical temperature (*T_c_*) and depairing current density (*J_d_*). GaP quantum dots with varying luminescence intensities were synthesized via the hot-injection method and subsequently introduced into the B(P)SCCO superconducting matrix. XRD and SEM characterization confirmed that the modified samples maintained high phase purity of the (Bi,Pb)-2223 structure along with a homogeneous microstructure. Electrical transport measurements revealed a dual, competing effect arising from the incorporated quantum dots: acting as a heterogeneous phase, they inherently generate an impurity effect that suppresses superconducting properties, while their electroluminescent characteristics potentially induce an enhancing effect. With the systematic increase in the luminescence intensity of the GaP quantum dots and optimization of their addition content, the superconducting performance of B(P)SCCO exhibited a corresponding upward trend, achieving an enhancement of approximately 1 K in *T_c_* and up to 20% in *J_d_* under optimal conditions. This work demonstrates a positive correlation between electroluminescent properties and superconducting parameters, establishing the potential feasibility of modulating superconducting performance through the controlled introduction of luminescent heterophases and thereby proposing a novel design strategy for the functionalization of superconducting materials.

## Figures and Tables

**Table 1:** Electroluminescence intensity and addition content of the luminescent hetero-phase of the samples S0 to S4.

Sample	Hetero-Phase	EL Intensity a.u.	Addition Content wt.%	Sintering Process
S0	/	/	0	840 °C 120 h
S1	GaP	/	0.2	840 °C 120 h
S2	GaP (1:1.4:1.4)	2100	0.2	840 °C 120 h
S3	GaP (1:1:1)	2950	0.15	840 °C 120 h
S4	GaP (1:1:1)	2950	0.2	840 °C 120 h

**Table 2:** Lattice parameters of (Bi,Pb)-2223 and phase volume fractions of the samples S0 to S4.

Sample	Lattice Parameters of (Bi,Pb)-2223				Phase Volume Fractions		
	a (Å)	b (Å)	c (Å)	V (Å^3^)	2223%	2212%	2201%
S0	5.41	5.42	37.12	1089.8	94.49	2.41	3.1
S1	5.41	5.41	37.10	1086.0	93.35	3.45	3.20
S2	5.41	5.42	37.12	1089.1	92.49	5.27	3.24
S3	5.41	5.42	37.12	1089.0	95.39	2.21	2.4
S4	5.41	5.42	37.12	1080.1	94.16	3.64	2.2

**Table 3:** Critical transition temperature *T_c,_*_0_, *T_c,on_*, *T_c_*, and room temperature resistance *R*_295*K*_ of the samples S0 to S4.

Sample	*T_c,_*_0_ (K)	*T_c,on_* (K)	*T_c_* (K)	Δ*T* (K)	*R*_295*K*_ (mΩ)
S0	103 ± 0.14	114 ± 0.12	107 ± 0.2	11	6.30
S1	95 ± 0.17	112 ± 0.13	104 ± 0.18	17	11.90
S2	97 ± 0.11	113 ± 0.16	105 ± 0.14	16	10.97
S3	98 ± 0.15	114 ± 0.18	106 ± 0.15	16	10.32
S4	102 ± 0.19	114 ± 0.14	109 ± 0.16	12	11.73

## Data Availability

The original contributions presented in this study are included in the article. Further inquiries can be directed to the corresponding author.
